# Characterization of Coastal Urban Watershed Bacterial Communities Leads to Alternative Community-Based Indicators

**DOI:** 10.1371/journal.pone.0011285

**Published:** 2010-06-23

**Authors:** Cindy H. Wu, Bram Sercu, Laurie C. Van De Werfhorst, Jakk Wong, Todd Z. DeSantis, Eoin L. Brodie, Terry C. Hazen, Patricia A. Holden, Gary L. Andersen

**Affiliations:** 1 Ecology Department, Earth Sciences Division, Lawrence Berkeley National Laboratory, Berkeley, California, United States of America; 2 Donald Bren of School of Environmental Science and Management, University of California Santa Barbara, Santa Barbara, California, United States of America; University of Wisconsin-Milwaukee, United States of America

## Abstract

**Background:**

Microbial communities in aquatic environments are spatially and temporally dynamic due to environmental fluctuations and varied external input sources. A large percentage of the urban watersheds in the United States are affected by fecal pollution, including human pathogens, thus warranting comprehensive monitoring.

**Methodology/Principal Findings:**

Using a high-density microarray (PhyloChip), we examined water column bacterial community DNA extracted from two connecting urban watersheds, elucidating variable and stable bacterial subpopulations over a 3-day period and community composition profiles that were distinct to fecal and non-fecal sources. Two approaches were used for indication of fecal influence. The first approach utilized similarity of 503 operational taxonomic units (OTUs) common to all fecal samples analyzed in this study with the watershed samples as an index of fecal pollution. A majority of the 503 OTUs were found in the phyla *Firmicutes*, *Proteobacteria*, *Bacteroidetes*, and *Actinobacteria*. The second approach incorporated relative richness of 4 bacterial classes (*Bacilli*, *Bacteroidetes*, *Clostridia* and *α-proteobacteria*) found to have the highest variance in fecal and non-fecal samples. The ratio of these 4 classes (BBC∶A) from the watershed samples demonstrated a trend where bacterial communities from gut and sewage sources had higher ratios than from sources not impacted by fecal material. This trend was also observed in the 124 bacterial communities from previously published and unpublished sequencing or PhyloChip- analyzed studies.

**Conclusions/Significance:**

This study provided a detailed characterization of bacterial community variability during dry weather across a 3-day period in two urban watersheds. The comparative analysis of watershed community composition resulted in alternative community-based indicators that could be useful for assessing ecosystem health.

## Introduction

Given that water sustains life, it is not surprising that a large percentage of the world's population lives near coastal regions [1], [2]. Coastal urban watersheds in the United States offer aesthetics and recreational value, serve as catchments for storm runoff, establish biological corridors for movements of wildlife, and provide buffers between developed areas and downstream waterways. As human populations increase, so does urbanization and lasting anthropogenic affects on creeks and coastal ecosystems [3]. According to a USEPA report (2007), 45% of streams and rivers, and 32% of bays and estuaries are impaired in the United States. Sources of impairment include pathogens and sewage discharges [4]. The presence of bacterial pollutants warrants comprehensive bacteriological characterization of these water bodies in order for us to understand their fate and transport in the environment.

Since pathogens often come from fecal sources, regulatory agencies require monitoring fecal indicator bacteria (FIB) for water quality assessments. Culture-dependent assays such as total coliform, fecal coliform and enterococci, and culture-independent assays such as quantitative PCR (qPCR) for *Bacteroides* and *Bifidobacterium* spp. [5] have been used as proxies for fecal pollution. However, enumeration of these indicator organisms often does not accurately represent the health of the ecosystem or associated risk [6] as these indicators are ubiquitous, persistent, regenerative [7], [8] and have low correlations with pathogen survival [9], [10] in the environment. Reliance upon single, even source-specific, markers of fecal pollution can be ineffective if they are labile or persistent relative to pathogens. The use of multiple indicators for tracking fecal contamination could circumvent the problem of single marker absence or presence and strengthen overall diagnoses of microbiological water quality [6], [7], [8], [9], [11].

With the advent of high throughput culture-independent characterization of microbial communities, such as microarray and sequencing approaches [12], [13], [14], [15], [16], detailed studies of bacterial community fluctuations due to physical, chemical and biological influences are now feasible. One such phylogenetic microarray, the PhyloChip, targets much of the known diversity within Bacteria and Archaea, and has been employed in a number of complex environments and conditions [17], [18], [19], [20], [21], [22], [23], [24], [25]. The current version (G2) of the PhyloChip provides the capability of identifying up to 8,741 Bacterial and Archaeal OTUs simultaneously [17], and allows for relative quantification of individual OTUs over a wide dynamic range [18], [26]. The highly parallel and reproducible nature of this array allows tracking community dynamics over time and treatment.

Bacterial communities in urban watersheds are sensitive to environmental perturbations and could provide information on impacts of fecal influence and overall ecosystem health. It is important to monitor the conditions of these watersheds because they are intricately tied in with downstream waterways, which could have public health risk and economic implications. Previous studies monitoring FIB most probable numbers (MPN) in urban creeks have found high temporal variability even during dry weather [27], [28], [29]. In Santa Barbara, California, exfiltration from sewer lines into the storm drain systems has been suspected to cause the observed high densities of FIB and human-specific *Bacteroides* markers (HBM) in urban watersheds that discharge into a recreational beach [29]. Here we analyze whole bacterial communities from the same Sercu et al. [29] samples in order to gain insights regarding the temporal and spatial dynamics of urban watershed bacterial community composition relevant to fecal pollution. Amplified 16S rRNA gene sequences from creek (including storm drains), lagoon and ocean sites in the Lower Mission Creek and Laguna watersheds in Santa Barbara, CA, along with 3 samples of fecal origin, were hybridized onto the PhyloChip for a complete microbial community analysis. Characterization of the whole bacterial community is crucial for understanding fluctuations of various bacterial groups, and could lead to more robust health risk indication by integrating data from multiple bacteria taxa. This work represents the first application of a comprehensive phylogenetic array for the purpose of characterizing urban watershed bacterial communities. Findings from this work suggest that such an approach could be useful for complementing multiple individual tests that are now typically employed to diagnose microbiological water quality related to public health.

## Results

### Resolving community differences by habitats

**Figure 1:**
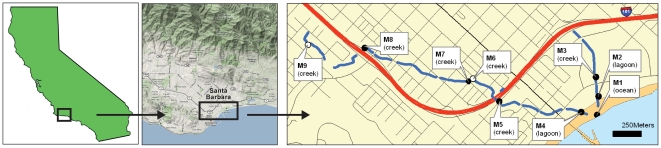
Sampling sites along Mission (M4–M9) and Laguna Channel (M2 and M3) watersheds. Samples were delineated into different habitat types: creek (M3, M5–M9, where M6 and M9 were from drains), lagoon (M2 and M4), and ocean (M1). Open circles (○) represent storm drains, and filled circles (•) represent creek, lagoon or ocean sites.

**Figure 2:**
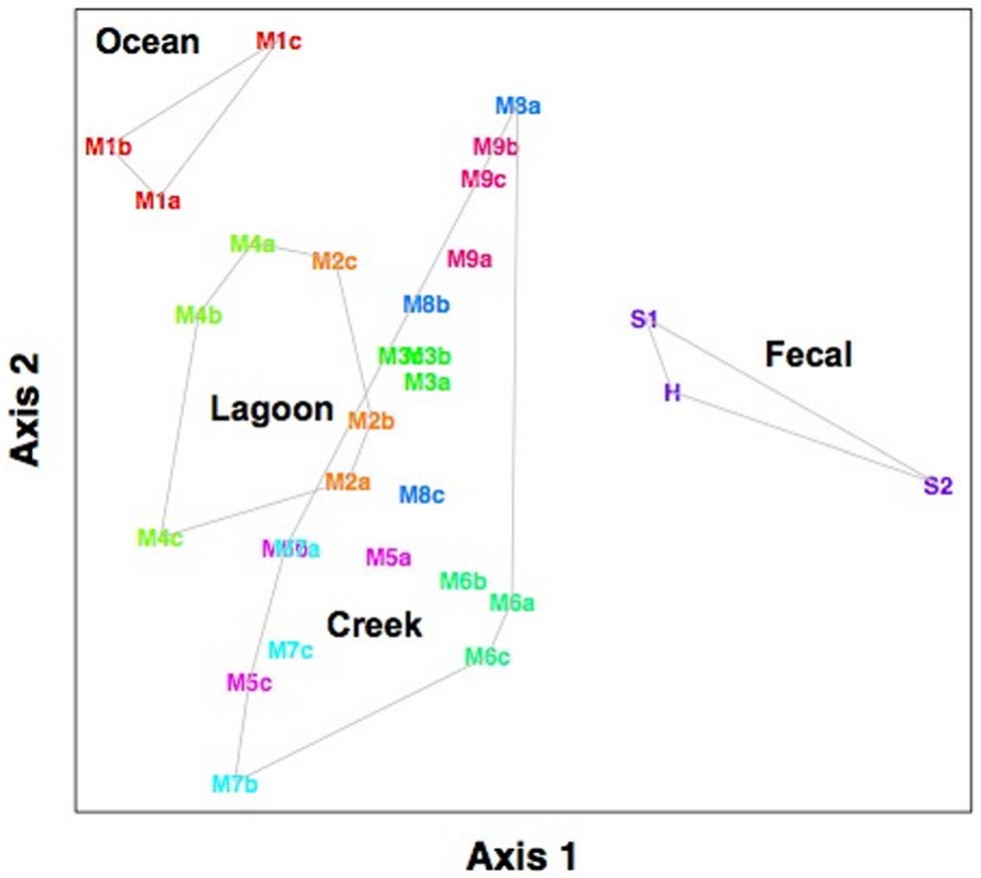
NMDS plot of PhyloChip community distances. Bray-Curtis metric was used, and a stress of 8.14 was obtained. Each site is represented by a different color. The grey lines delineate grouping of creek, lagoon, ocean and fecal samples.

Samples were categorized into 4 habitat types: fecal, ocean, lagoon, and creek (Figure 1). Comparisons of Bray-Curtis distances of the communities, using Multi-Response Permutation Procedure (MRPP) [30], indicated significant differences between the samples from the different habitat types. Non-metric multidimensional scaling (NMDS) ordination illustrated that the bacterial communities were separated by habitat types for most of the samples, except for M2a and M2b (Figure 2). Salinity measurements at one of the lagoon sites (M2) were low, at ∼1 ppt, on days 1 (M2a) and 2 (M2b) (Table S1). On day 3 (M2c), the salinity increased to 5.3 ppt, and a corresponding community composition shift was observed (Figure 2). The bacterial communities of M2a and M2b were more similar to creek samples with low salinity and M2c was more similar to the M4a and M4b lagoon samples, which had higher salinity measurements of 7.3–9.5 ppt. Lagoon sample M4c had lower salinity measurements and the community was more similar to creek samples than to M4a and M4b.

**Figure 3:**
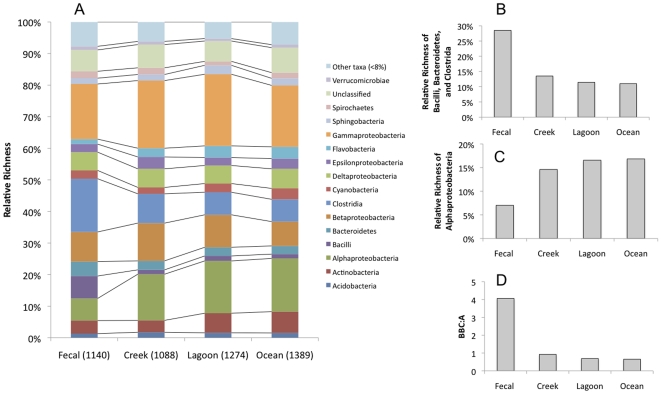
Bacterial community composition comparison across fecal, lagoon, creek and ocean samples. (A) Distribution of relative richness at the class level. Number of OTUs in each sample types were divided by the total count for each sample type as indicated in parentheses on the x-axis. (B) Relative richness of *Bacilli*, *Bacteroidetes* and *Clostridia* detected. (C) Relative richness of α-pro*teobacteria* detected. (D) *Bacillus*, *Bacteroidetes*, *Clostridia* to *α-proteobacteria* ratios (BBC∶A).

Distributions of detected operational taxonomic units (OTUs) at the class level were compared among all habitat types, shown as relative richness (Figure 3A). The relative richness was normalized to the total number of OTUs detected in all of the samples from the same habitat type. We focused on classes that exhibited high variability of relative richness across the 4 habitats. The top 10 classes with the highest standard deviations were (in descending order): *Clostridia*, *α-proteobacteria*, *Bacilli*, *γ-proteobacteria*, *β-proteobacteria*, *Actinobacteria*, *Flavobacteria*, *Bacteroidetes*, *Cyanobacteria* and *ε-proteobacteria*. Of those classes, only *Clostridia*, *Bacilli*, and *Bacteroidetes* had higher relative richness in fecal samples than in creek, lagoon and ocean samples (Figure 3B). Only *α-proteobacteria* had lower richness in fecal samples than in creek, lagoon, and ocean samples (Figure 3C). The characteristics and potential of these 4 classes as indicators of fecal influence will be discussed further.

### Fecal sample-associated OTUs

In order to define bacteria that were common to all 3 fecal samples used in this study, a set of 503 OTUs, found in all fecal samples but not ubiquitous in the 27 watershed samples, were characterized and defined as fecal sample-associated OTUs (FSAO). The FSAO subpopulation consisted of 43% *Firmicutes* (out of the 503 OTUs), 28% *Proteobacteria*, 9% *Bacteroidetes* and 5% *Actinobacteria* (Figure S1). Of the *Firmicutes* (218 OTUs), 56% were from the order *Clostridiales* including the families *Lachnospiraceae*, *Peptostreptococcaceae*, *Peptococcaceae*, *Acidaminococcaceae* and *Clostridiaceae*; 17% were from the order *Bacillales* including *Bacillaceae*, *Halobacillaceae*, and *Staphylococcaceae*; and 17% were from *Lactobacillales* which included the families of *Lactobacillaceae*, *Enterococcaceae* and *Streptococcaceae*. In the *Proteobacteria* phylum (141 OTUs), 30% were from *Enterobacteriales* including *Enterobacteriaceae*; 7% were from *Alteromonadales* including *Alteromonadaceae*, and *Shewanellaceae*; 8% of the OTUs were from the order *Burkholderiales* including *Burkholderiaceae*, *Comamonadaceae*, *Alcaligenaceae*, *Oxalobacteraceae*, and *Ralstoniaceae*. The counts of FSAO for each of the three days are shown in Figure S2. The FSAO counts were highest at M9, M8, M6, M3 and M2 and lowest at M4 and M1. The 3-day average FSAO counts for sites M9, M6, M3, and M2 were significantly different (*t*-test, p-value<0.0001) from counts of M4, and M1.

### Variable and stable subpopulations

**Figure 4:**
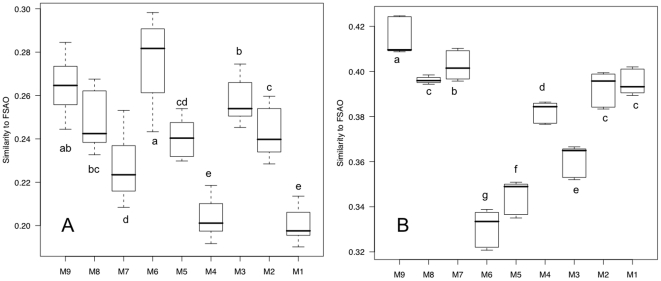
Boxplots of UniFrac similarity metrics between water and fecal-sample-associated OTUs (FSAO). (A) Variable subpopulations. (B) Stable populations. Each box represents similarity metrics from all 3 days at each site. Boxplots with different letters indicate significant differences (p-value<0.05), compared using the student *t*-test. The samples were arranged from upstream to downstream (left to right) for samples M9-M4, and M3-M2.

PhyloChip analysis of subpopulations from each site for which the fluorescence intensities fluctuated the most (variable) and the least (stable) were examined over the course of the three-day sampling period. These variable and stable subpopulations consisted of OTUs from the top and bottom deciles after sorting based on variance of fluorescence intensity over the 3 days. A similarity metric, from the UniFrac [31] distance measure, was illustrated with boxplots for comparison of the median, upper and lower quartiles. Variable subpopulations of M6 were the most similar to the FSAO composition in comparison to the other sites (Figure 4A). Sites M9 and M3 were the second and third most similar to the FSAO. However, the similarity to FSAO for site M9 was not significantly different from that of M6 or M3. A pattern of decreasing similarity from M9, M6 and M3 to immediate downstream sites was illustrated. The majority of FSAO detected in the variable subpopulations was in the orders of *Enterobacteriales* (39 out of 58 FSAO detected in the variable subpopulation) for M6, *Campylobacterales* (6 out of 44) for M9, and *Flavobacteriales* (4 out of 31) for M3. The M9 stable subpopulation was the most similar to the FSAO, and was significantly different from the similarity to FSAO of all other sites (Figure 4B). Many of the FSAO in the M9 stable subpopulation were in the order of *Bacillales* (17 out of 47).

### Ratio of *Bacilli*, *Bacteroidetes* and *Clostridia* to *α-proteobacteria*

**Figure 5:**
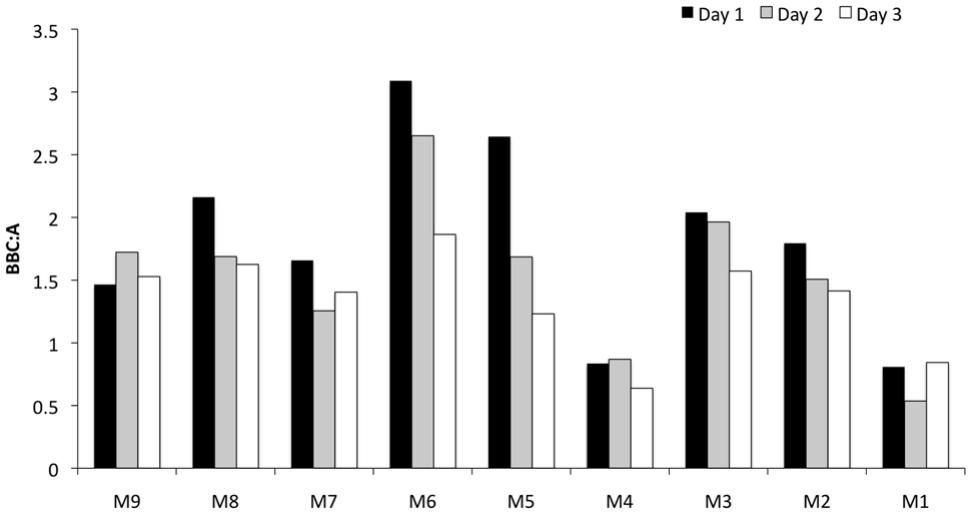
*Bacillus*, *Bacteroidetes*, *Clostridia* to *α-proteobacteria* ratios (BBC∶A) from each site. Ratio from each day is represented by a bar of different color.

Four bacterial classes, which exhibited highly fluctuating relative richness across the habitat types, were further explored as representatives of the fecal bacterial community (Figure 3A). The combined percentage of *Bacilli*, *Bacteroidetes and Clostridia* relative richness was 28.5% of total detected in the fecal samples, whereas in creek, lagoon and ocean they were less than 13.5% (Figure 3B). Almost 15% of the relative richness in creek water, lagoon and ocean samples were *α-proteobacteria*, while the percentage of *α-proteobacteria* found in fecal samples was 7% (Figure 3C). The relative richness ratio of *Bacilli*, *Bacteroidetes and Clostridia* to *α-proteobacteria* (BBC∶A) for fecal samples was more than 4-fold higher than the ratios of the other habitat types (Figure 3D). The BBC∶A ratio was calculated for each of the samples from the different sites (Figure 5). Site M6 exhibited the highest BBC∶A, and sites M1 and M4 had low BBC∶A ratios compared to the rest of the sites.

### Retrospective comparison of BBC∶A ratios from 16S rRNA gene clone library sequencing- and PhyloChip-analyzed samples

**Figure 6:**
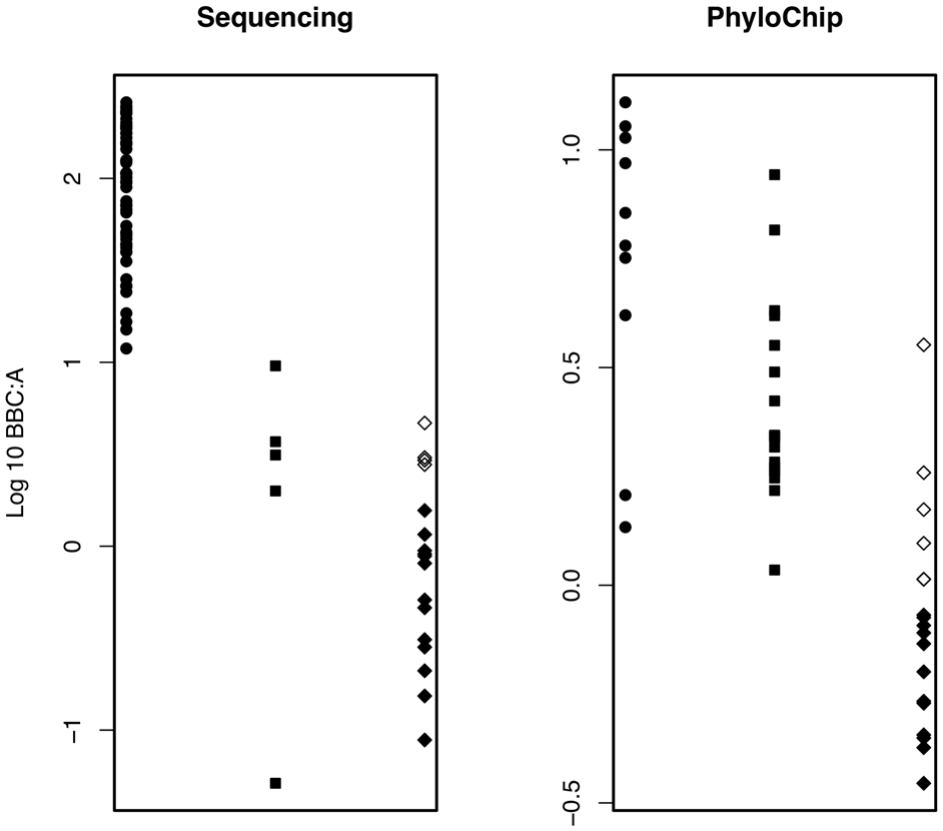
*Bacillus*, *Bacteroidetes*, *Clostridia* to *α-proteobacteria* ratios (BBC∶A) of communities analyzed by sequencing or PhyloChip. Sample types include, gut (•), sewage-associated (▪), and non-fecal (♦) associated samples. Unfilled diamond symbols (⋄) represent non-fecal samples from anoxic environments.

The BBC∶A ratios of 124 communities characterized by clone-library sequencing and PhyloChip were compared (Figure 6). Detailed descriptions of the communities are included in Table S2. From published sequencing studies, we calculated the BBC∶A ratios of bacterial communities from 54 mammalian intestines [32], 5 sewage-associated samples [33], [34], [35], [36], [37], and 19 non-fecal samples [23], [38], [39], [40], [41], [42], [43], [44], [45], [46], [47], [48], [49], [50], [51], [52], [53]. Likewise, from PhyloChip-analyzed samples, we determined the BBC∶A ratios from communities of 11 gut [Brodie et al., unpublished; Marchesi et al., unpublished; Nguyen et al., unpublished; This study] [54], 17 sewage-associated [Conrad et al., unpublished; Sercu et al. unpublished; Wu et al., unpublished], and 18 non-fecal samples [Sercu et al., unpublished; This study] [55]. Anoxic non-fecal samples were included in this comparison as well. For both PhyloChip- and library sequencing-analyzed bacterial communities, gut and sewage-associated samples generally had higher BBC∶A ratios than non-fecal samples, except for anoxic non-fecal samples, which had an overlapping range with sewage-associated samples. There were also a few communities that did not follow the general BBC∶A ratio trend. The community of a nitrifying-denitrifying activated sludge [35] had much lower BBC∶A ratio than the rest of the sequenced sewage-associated communities. Also, beetle posterior hindgut and midgut communities had lower BBC∶A ratios than beetle anterior hindgut communities and the other PhyloChip-analyzed gut samples [Nguyen et al., unpublished].

## Discussion

Microbial communities in surface waters are highly responsive to perturbation, shifting with tidal cycles [56], salinity gradients [57], [58], dissolved organic matter concentration [59], and chemical stress [60], [61], [62]. The detection of short-term fluctuations in community composition suggests changes in environmental conditions, nutrients or bacterial sources. An effect of increased salinity due to tidal influence on bacterial composition was observed in this study where the coastal lagoon communities were more similar to creek communities with comparable salinity measurements (Figure 2). Salinity was more strongly correlated to community composition than the other environmental variables measured based on canonical correspondence analysis (data not shown). This result corroborated observations by others [63], [64], [65]. In addition to being highly sensitive to environmental fluctuations, the response time of community composition shift was within a 24-hour period.

The detection of this rapid community response could be useful for indication of external bacterial inputs, such as from fecal sources. FSAO, derived from the human fecal and untreated sewage samples, were used to represent fecal communities. One caveat is that the OTUs in the FSAO list are specific to the 3 fecal samples used in this study, and do not represent all fecal communities in all environments. However, the prevalent bacterial phyla found in the FSAO are the same as those observed in published studies of human gastrointestinal tract samples [66], [67], [68], [69] and turkey cecal samples [70]. Therefore, community similarity to FSAO could potentially indicate the presence of fecal bacteria. This hypothesis was tested by comparing the community distances between FSAO and variable/stable subpopulations at each of the site (Figure 4A and 4B).

Examination of the variable and stable subpopulations brings to light the bacterial temporal fluctuations across the 3 days. The variable subpopulation represents OTUs with highly fluctuating relative abundances, perhaps due to rapid growth, decay or large sporadic influx of bacterial sources. The stable subpopulation represents OTUs with constant relative abundances. These stable subpopulation OTUs are likely associated with endemic bacteria that are able to grow and persist under the *in situ* environmental conditions or are from consistent external sources.

UniFrac analysis showed that the variable subpopulation of M6 was the most similar to the FSAO (Figure 4A). This suggested intermittent exposure to fecal sources at this site, which was supported by elevated but numerically variable HBM densities and FIB MPN (Figure S3). The prevalence of *Enterobacteriales* in the variable subpopulation falls in line with the high FIB MPN observed at site M6, and further supports the use of similarity of the variable subpopulation with FSAO for demonstrating fecal pollution. Similarity of M9 variable subpopulation to FSAO was not significantly different from that of the M6 (Figure 4A). This indicated that there were OTUs in the M9 variable subpopulation that were also found in the FSAO, but they were mostly from the order of *Campylobacterales*, and not represented by FIB or HBM detection. The similarity to FSAO decreased gradually from drains to downstream sites (i.e. M9 to M7 and M6 to M4), illustrating possible fecal community presence at the drains and die-off or dilution effects as the communities flow downstream.

Interestingly, the stable subpopulation at M9 was most similar to FSAO out of all the sites, even though the FIB densities met the California water quality standards on 2 out of the 3 days and no HBM was detected (Figure 4B and Figure S3). The non-detection of HBM at M9 could be due to *Bacteroides* DNA concentration being below the quantitative PCR detection limit of 0.5×10^3^–10^4^ targets L^−1^ [29] or that the fecal source was non-human. The top three families present in the M9 stable subpopulation were *Bacillaceae*, *Staphylococcaceae* and *Lachnospiraceae*. While *Bacillaceae* and *Staphylococcaceae* have been observed in non-aquatic environments [22], [26], *Lachnospiraceae* are primarily associated with cow rumen [71], human bowel [67] and anaerobic digesters [72]. Therefore, the data suggested that some of the OTUs detected at M9 could have a fecal, but non-human, origin. However, further confirmatory work is needed to distinguish between a consistent fecal source or bacterial re-growth as the cause for the similarity between M9 stable subpopulation and FSAO.

The FSAO includes OTUs that contain fecal coliforms, which have been demonstrated to re-grow and persist in the environment leading to false-positive water quality diagnoses [6], [8], [73]. This study further explores the potential of using alternative organisms that are independent of coliforms as fecal indicators by introducing the BBC∶A ratio. The ratio excludes coliform bacteria, thus, potentially avoids false-positive results associated with coliforms, and integrates counts for organisms widespread in non-fecal “pristine” environments to assess ecosystem health.

*Bacteroidetes* and *Clostridia* are enriched within the gut microbiota of many mammals [32], [66], [67], [68], [69], [70], and specific species within these 2 classes have been proposed as fecal indicators [5], [10], [74]. However, they are also found in anoxic saline aquatic environments [40], [45], [49], estuaries [38], the deep ocean [41], and high elevation lakes [59]. The class of *Bacilli*, which includes the indicator species *Enterococcus*, is commonly found in fecal samples such as the human gastrointestinal tract [69], turkey intestines [69], [70] and aerobic thermophilic swine wastewater bioreactors [75]. All 3 classes are dominant groups found in a chicken fecal metagenomic study [76]. α-pro*teobacteria*, have been found as primary surface colonizers in coastal marine waters [77] and have the ability to thrive under low-nutrient conditions [56]. The BBC∶A ratio incorporates the relative richness of OTUs prevalent in these 4 bacterial classes associated with fecal and non-fecal samples to reflect possible fecal inputs, rather than the use of single organism presence or absence. Previous studies have suggested the use of ratios for indicating human or non-human fecal pollution [78], determining fecal age and enteric viral content [79], [80], representing the nutrient status of soil ecosystems [81], [82], identifying land use in wetland soils [83], and eutrophy in aquatic systems [84].

In order to assess the applicability of the observations from our watersheds to other samples, we calculated the BBC∶A ratio from previously published and unpublished studies (Table S2). BBC∶A ratios of gut samples analyzed by DNA sequencing or PhyloChip are not completely comparable, mainly due to differences in sample processing including primers used, PCR conditions and coverage differences across phylogenetic groups on the PhyloChip. However, within communities analyzed by sequencing from different research groups employing varying protocols, the gut, sewage-associated and non-fecal samples exhibited the same BBC∶A ratio trend as those communities analyzed by PhyloChip processed with a consistent standardized protocol. The distribution of BBC∶A ratios from these studies illustrates that gut and sewage-associated samples have higher BBC∶A ratio than non-fecal samples regardless of analysis methods (Figure 6). Anoxic non-fecal polluted environments also have similar ratios of BBC∶A as sewage-associated samples (Figure 6). This is most likely an attribute of similar growth conditions favoring both anaerobic and fecal bacteria. The indication of anoxic non-fecal environments is often times pertinent for determining public health risks. Anoxic conditions could lead to eutrophication in both fresh and salt water environments, which changes nutrient cycling, water quality and biodiversity [84]. Eutrophication has led to toxic algal blooms that adversely affect human and wildlife health [85], [86].

Kendall rank correlation of FIB, HBM, FSAO and BBC∶A ratios from all sites indicated significant positive correlations of BBC∶A ratios with HBM, total coliform, enterococcus and FSAO counts, but not with *E. coli* (Table S3). However, many of the samples had reached the total coliform measurement maximum detection limit of 24,196 MPN, therefore, the correlation of total coliform with BBC∶A ratio might be misleading. The result also illustrated that even though the BBC∶A ratio did not contain fecal coliforms, the fecal pollution pattern was similar to that indicated by the FSAO where coliforms were included. The drain site M6 was the only site where all lines of evidence, i.e. similarity of variable subpopulations to FSAO, FIB, HBM, and BBC∶A ratios, pointed to the presence of fecal contamination. At site M1 (ocean), all data indicated a community with the least fecal influence. The data for the rest of the sites (M2, M3, M4, M5, M7, M8 and M9) indicated varying degrees of influence by fecal sources. Also, communities from drains (M6 and M9) were the most similar to organisms found in the fecal samples, although different fecal organisms were detected in the two drains.

Knowledge of who is there and how they change over time and location is the hallmark of an ecosystems approach to studying urban watersheds. We used this concept to track the microbial community dynamics over a three-day period at a location with a history of frequent fecal contamination. In spite of the confounding effect of the movement of water through this watershed, several patterns that correlated with the presence of human fecal contamination were observed. By using the PhyloChip we are able to identify a significantly greater number of bacterial OTUs than is typically examined in coastal watersheds. Comparison of the microbial inventory of the watershed samples with local sewage samples and a human fecal sample led to the identification of specific organisms that were associated with either potential human fecal sources or with the watershed. From this information we observed 503 OTUs that were common to the three fecal samples (FSAO) and the ratios of observed classes of organisms that demonstrated the largest differences between human fecal sources and the receiving waters (BBC∶A ratio). Whereas most research for measuring fecal influences on coastal watersheds uses a bottom-up approach to hypothesize that a specific organism is representative of the source, we employed a top-down approach that looked at a large number of potential bacterial contaminants from a majority of the known bacterial diversity to identify a diverse collection of organisms associated with fecal pollution. The advantage of this approach is that we can use the findings of the BBC∶A ratio and the FSAO as the basis for additional bottom-up, controlled experiments to examine their applicability at other locations and with other human fecal sources. Using this more detailed microbial community characterization, it may be possible to move away from generic, single indicators to a community-indicator approach for assessing fecal contamination or environments conducive to pathogen growth.

## Materials and Methods

### Ethics statement

The Human Subjects Committee of University of California, Santa Barbara was informed of the anonymous human sample used in this study, and declared that the sample did not meet the definition of a human subject sample, therefore, no approval was necessary for it's use.

### Sample description, collection and extraction

Mission Creek and Laguna Channel flow through an urbanized area of downtown Santa Barbara and discharge at a popular bathing beach. As described previously [29], water column samples from 3 consecutive days (a = day 1, b = day 2, c = day 3), during the dry season (June 2005), were collected from 9 locations (M1–M9) within the Mission Creek and Laguna watersheds in Santa Barbara, California (Figure 1). Samples were delineated into different habitat types: creek (M3, M5–M9, where M6 and M9 were from drains), lagoon (M2 and M4), and ocean (M1). One sample per day was collected at approximately the same time on each of the 3 days. No rain occurred at least 48 hours prior to or during the sampling. The creek flow rate, taken at M5, was 0.016 m^3^s^−1^. Both watersheds discharged into the same lagoon at M2 and M4. Surface water flowed from the lagoon into the ocean (M1) at the time of sampling. Three fecal samples, 1 human feces (H), from Santa Barbara, and 2 raw sewage, from the influent at El Estero Wastewater Treatment plant (Santa Barbara, CA) (S1, S2), were also collected. Dissolved oxygen (DO), pH, temperature and salinity were measured along with each sampling [29]. Water samples were filtered in the lab onto 0.22 µm filters on the day of the sampling and stored at −20°C until nucleic acid extractions. DNA was extracted using the UltraClean Water DNA kit (MoBio Laboratories, Inc. Carlsbad, CA, USA), and archived at −20°C. Concentrations of fecal indicator bacteria (FIB) which includes total coliforms, *E. coli*, and *Enterococcus spp.*, and quantitative PCR (qPCR) measurements of Human-specific *Bacteroides* Marker (HBM) were reported previously [29].

### 16S rRNA gene amplification for microarray analysis

Genes encoding 16S rRNA were amplified from the gDNA using non-degenerate Bacterial primers 27F and 1492R [87]. Polymerase chain reaction (PCR) was carried out using the *TaKaRa Ex Taq* system (Takara Bio Inc, Otsu, Japan). The amplification protocol was previously described [17].

### Microarray processing, and image data analysis

Microarray analysis was performed using the PhyloChip, an Affymetrix-platform microarray. The protocols were previously reported [17]. Briefly, amplicons were concentrated to a volume less than 40 µl by isopropanol precipitation. The DNA amplicons were then fragmented with DNAse (Invitrogen, Carlsbad, CA, USA), biotin labeled, denatured, and hybridized to the DNA microarray at 48°C overnight (>16 hr). The arrays were subsequently washed and stained. Reagents, conditions, and equipments involved are detailed elsewhere [88]. Arrays were scanned using a GeneArray Scanner (Affymetrix, Santa Clara, CA, USA).

The CEL files obtained from the Affymetrix software that produced information about the fluorescence intensity of each probe were analyzed. The detailed criteria for scoring the probe fluorescence intensities were described elsewhere [17], [18], [89]. Briefly, a probe set consisted of 11 or more specific 25-mers (probes) that were prevalent in members of a given OTU but were dissimilar from sequences outside the given OTU. Probes with sequences complementing all 25 base pairs of the target sequences were termed perfect match (PM) probes. Each PM probe was matched with a control 25-mer, identical in all positions except the 13^th^ base, termed mismatch (MM) probe. The PM and MM constituted a probe pair that were analyzed together. The probe pairs were scored as positive if the following two criteria were met: 1) the intensity of fluorescence from the PM probe was greater than 1.3 times the intensity from the MM probe, and 2) the difference in intensity (PM minus MM), was at least 500 times greater than the squared noise value. The CEL files from this study are available upon request.

The taxonomic position of each OTU as well as the accompanying NCBI accession numbers of the sequences composing each OTU can be viewed in outline format at: http://greengenes.lbl.gov/Download/Taxonomic_Outlines/G2_chip_SeqDescByOTU_tax_outline.txt.

### PhyloChip data normalization

PhyloChip data normalization was performed using R [90]. To correct for variation associated with quantification of amplicon target (quantification variation), and downstream variation associated with target fragmentation, labeling, hybridization, washing, staining and scanning (microarray technical variation) a two-step normalization procedure was developed. First, for each PhyloChip experiment, a scaling factor best explaining the intensities of the spiked control probes under a multiplicative error model was estimated using a maximum-likelihood procedure [54]. The intensities in each experiment were multiplied with its corresponding optimal scaling factor. Second, the intensities for each experiment were corrected for the variation in total array intensity by dividing the intensities with its corresponding total array intensity separately for Bacteria and Archea. The normalized data is available in Table S4.

### Statistical Analysis

All statistical analyses were carried out in R [90], except for the canonical correspondence analysis (CCA). Bray-Curtis distances were calculated using normalized fluorescence intensity with the *ecodist* package [91]. Non-metric multidimensional scaling (NMDS) and multi response permutation procedure (MRPP) was performed using the *vegan* package. Student *t*-test and Kendall rank correlation from the *stats* package were used to compare samples. A relaxed neighbor-joining tree was generated using *Clearcut* [*92*] and used for UniFrac analysis [31]. Unweighted UniFrac distances, converted to similarity metrics, were calculated for FSAO, variable and stable subpopulations. CCA was carried out using PCOrd [93]. There were no DO, pH and salinity data for sampling days 1 and 2 for site 6, and all 3 days of sampling for site 8. No environmental variables were measured for fecal sample data. Therefore, best-estimate values were inserted based on values measured from the nearest sites on the same day for the CCA. Fecal sample environmental variables were estimated based on reported values in literature.

### PhyloChip derived parameters

Unless otherwise stated, an OTU was considered present when at least 90% of its assigned probe pairs for its corresponding probe set were positive (positive fraction ≥0.9). For example, if 10 out of 11 probe pairs are positive, the positive fraction is 0.909 and the OTU is considered present.

Fecal-sample associated OTUs (FSAO) - OTUs that were present in all 3 fecal samples, and in all 27 water samples were tabulated separately. The list of 503 FSAO was derived by removing those OTUs found in all 27 water samples from the OTUs that were present in the fecal samples. The OTUs in each sample which were also found on the list of 503 FSAO were tallied and presented as the FSAO count.

Variable and stable subpopulations - OTUs that were present in at least one of the 3 samples from each site were tabulated and variances of the fluorescence intensities across the 3 days for those OTUs were generated. The OTUs were sorted by variance in descending order. The OTUs in the top deciles (90^th^ percentile) were defined as the variable subpopulation, and OTUs in the bottom deciles (10^th^ percentile) were defined as the stable subpopulation.

The BBC∶A ratio of phyloChip samples - The number of OTUs in the classes of *Bacilli* (Bac), *Bacteroidetes* (Bct), *Clostridia* (Cls), and *α-proteobacteria* (A) where the positive fraction equal to 1 were tallied. The ratio was calculated using the following formula:
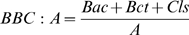
where
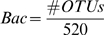


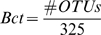


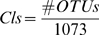


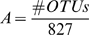
The count for unique OTUs in each of the class was normalized by dividing by the total number of OTUs in each class detectable by the G2 PhyloChip. The denominators were predetermined based on the number of OTUs assigned for each bacterial class on the G2 PhyloChip design.

### The BBC∶A ratio of published 16S rRNA gene clone library sequencing samples

Aligned sequences in the Greengenes [94] database were downloaded and re-classified using the PhyloChip (G2) taxonomy on the Greengenes website (http://greengene.lbl.gov). Aligned DNA sequences of various environmental communities were also obtained from [63]. The counts of unique OTUs were tallied for each bacterial class. The BBC∶A ratios were calculated using the formulas mentioned above. If no OTU was detected for that class, the count was set to 0.5.

## Supporting Information

Figure S1 Phylum level profile of 503 fecal sample-associated OTUs (FSAO), and order level profiles of Firmicutes and Proteobacteria. Pie chart illustrates that the FSAO consist of 43% Firmicutes and 28% Proteobacteria. Most of the Firmicutes OTUs are in the order of Clostridiales, and most of the Proteobacteria OTUs are in Enterobacteriales.
(0.31 MB TIF)

Figure S2 Counts of fecal-sample-associated OTUs (FSAO) at each site. Each bar represents one sample from each day. OTUs in each sample which were also found on the list of the 503 FSAO were tallied and presented as the FSAO count.
(0.15 MB TIF)

Figure S3 Measurements of Human-specific Bacteroides Marker (HBM), Total Coliform (TC), E. coli (EC), and Enterococcus (ENT) counts. Bars represent HBM values. Lines represent TC, EC and ENT most probable number (MPN).
(0.49 MB TIF)

Table S1 Environmental variables measured concurrently with the bacterial community samples. Dissolved oxygen, temperature, salinity and pH were measured at the time of sampling and reported here.
(0.42 MB TIF)

Table S2 Description of bacterial communities analyzed by sequencing and PhyloChip used in Figure 6. Gut, sewage-associated and non-fecal samples analyzed by clone-library sequencing and PhyloChip used for the *Bacilli*, *Bacteroidetes*, *Clostridia* to *α-proteobacteria* ratio (BBC∶A ratio) are described. All DNA sequences from sequencing samples had a minimum length of 1250 base pairs, except for those with the (*) symbol where the minimum sequence length was 200 base pairs.
(0.90 MB TIF)

Table S3 Kendall rank correlation tau coefficient and p-values (in parenthesis). Measurements from all 27 water samples were used. The (*) symbol denotes statistical significance (p-value<0.05) differences. Abbreviations: Human Bacteroides Marker (HBM); total coliform (TC); E. coli (EC); enterococcus (ENT); fecal-sample associated OTUs (FSAO); *Bacilli*, *Bacteroidetes*, and *Clostridia* to *α-proteobacteria* ratio (BBC∶A).
(0.09 MB TIF)

Table S4 Total OTUs detected by PhyloChip for all 30 samples. Positive fraction and normalized fluorescence intensity values are reported.
(3.87 MB XLS)
