# Supplementary figures and images for: Characterization of Coastal Urban Watershed Bacterial Communities Leads to Alternative Community-Based Indicators
Source: PLoS One. 2010 Jun 23;5(6):e11285. doi: 10.1371/journal.pone.0011285 (PMC2890573; doi:10.1371/journal.pone.0011285)

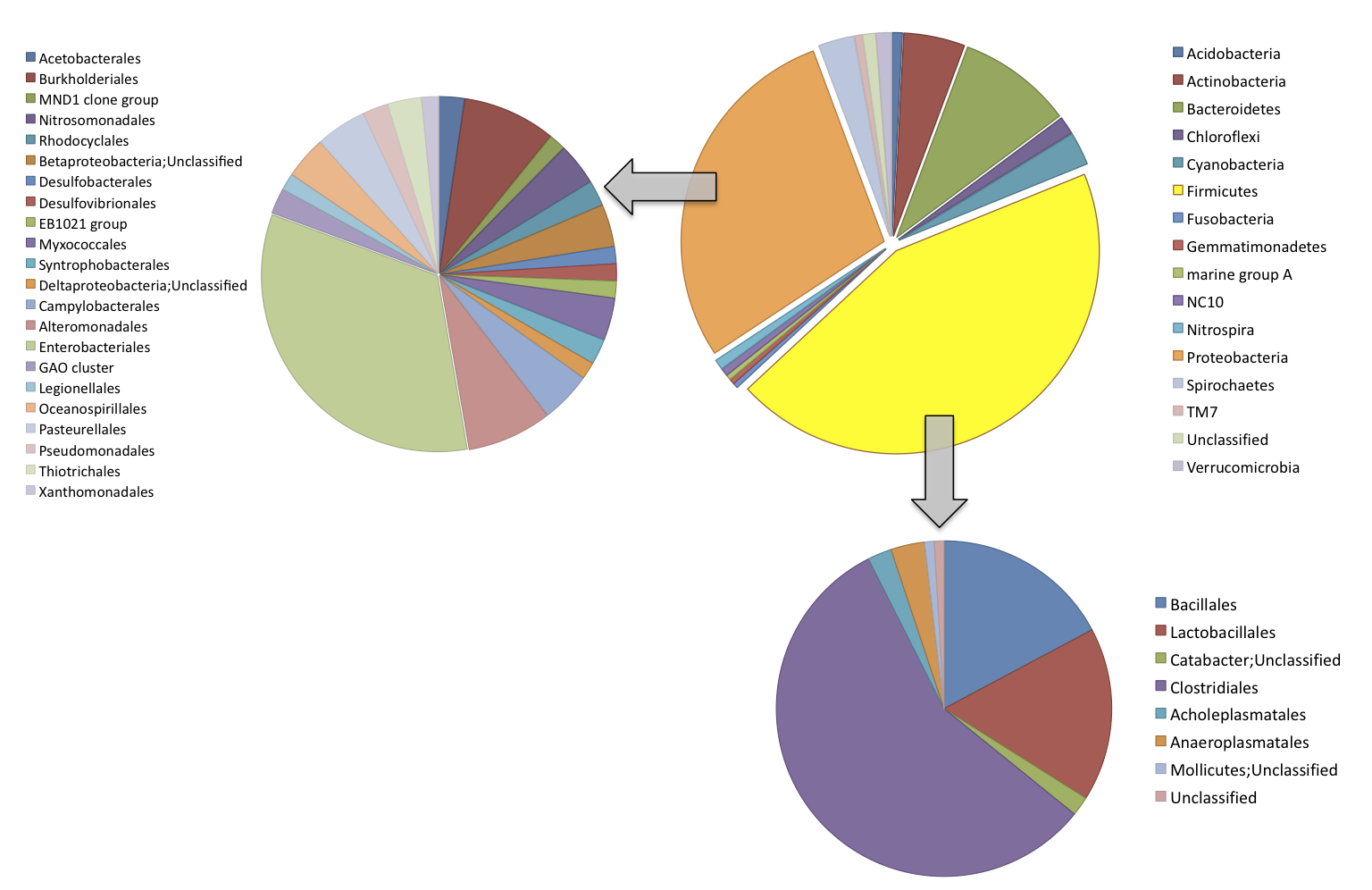

Supplement: Figure S1 — Phylum level profile of 503 fecal sample-associated OTUs (FSAO), and order level profiles of Firmicutes and Proteobacteria. Pie chart illustrates that the FSAO consist of 43% Firmicutes and 28% Proteobacteria. Most of the Firmicutes OTUs are in the order of Clostridiales, and most of the Proteobacteria OTUs are in Enterobacteriales. (0.31 MB TIF) [file pone.0011285.s001.tif]

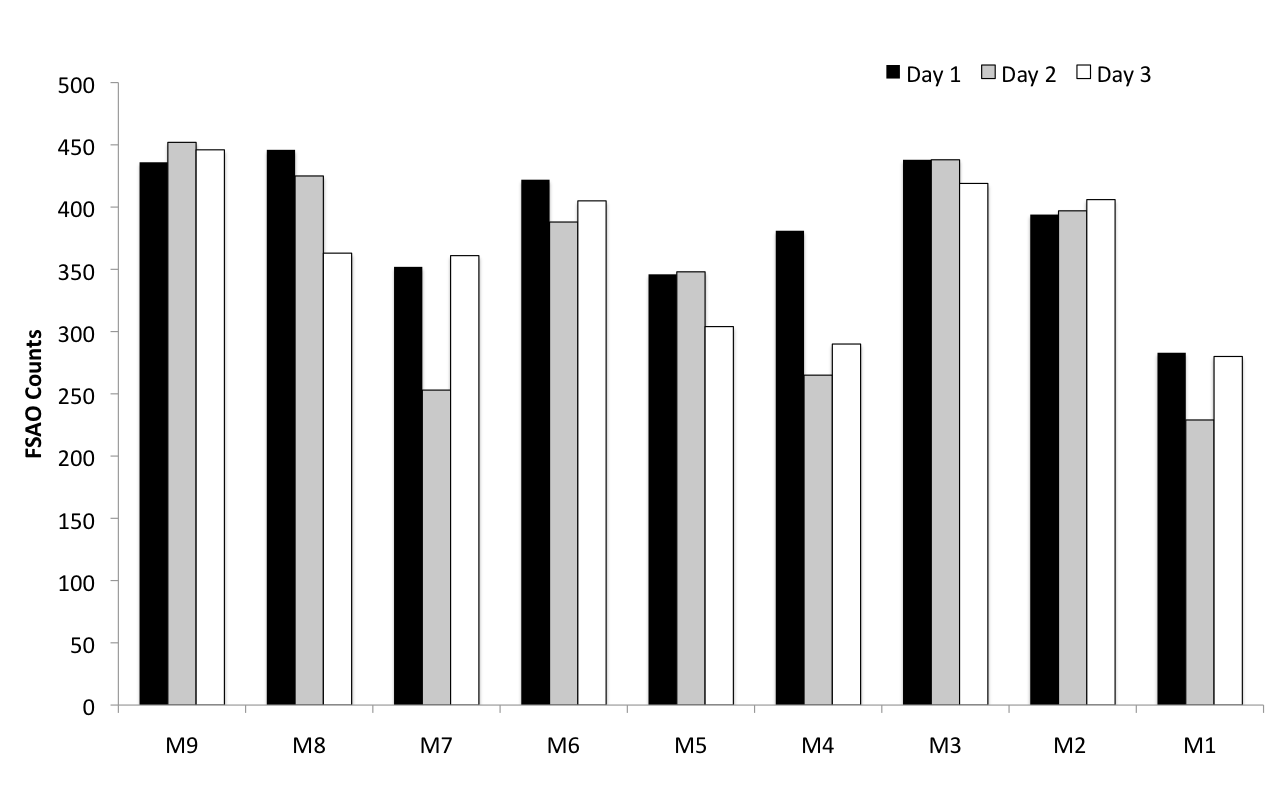

Supplement: Figure S2 — Counts of fecal-sample-associated OTUs (FSAO) at each site. Each bar represents one sample from each day. OTUs in each sample which were also found on the list of the 503 FSAO were tallied and presented as the FSAO count. (0.15 MB TIF) [file pone.0011285.s002.tif]

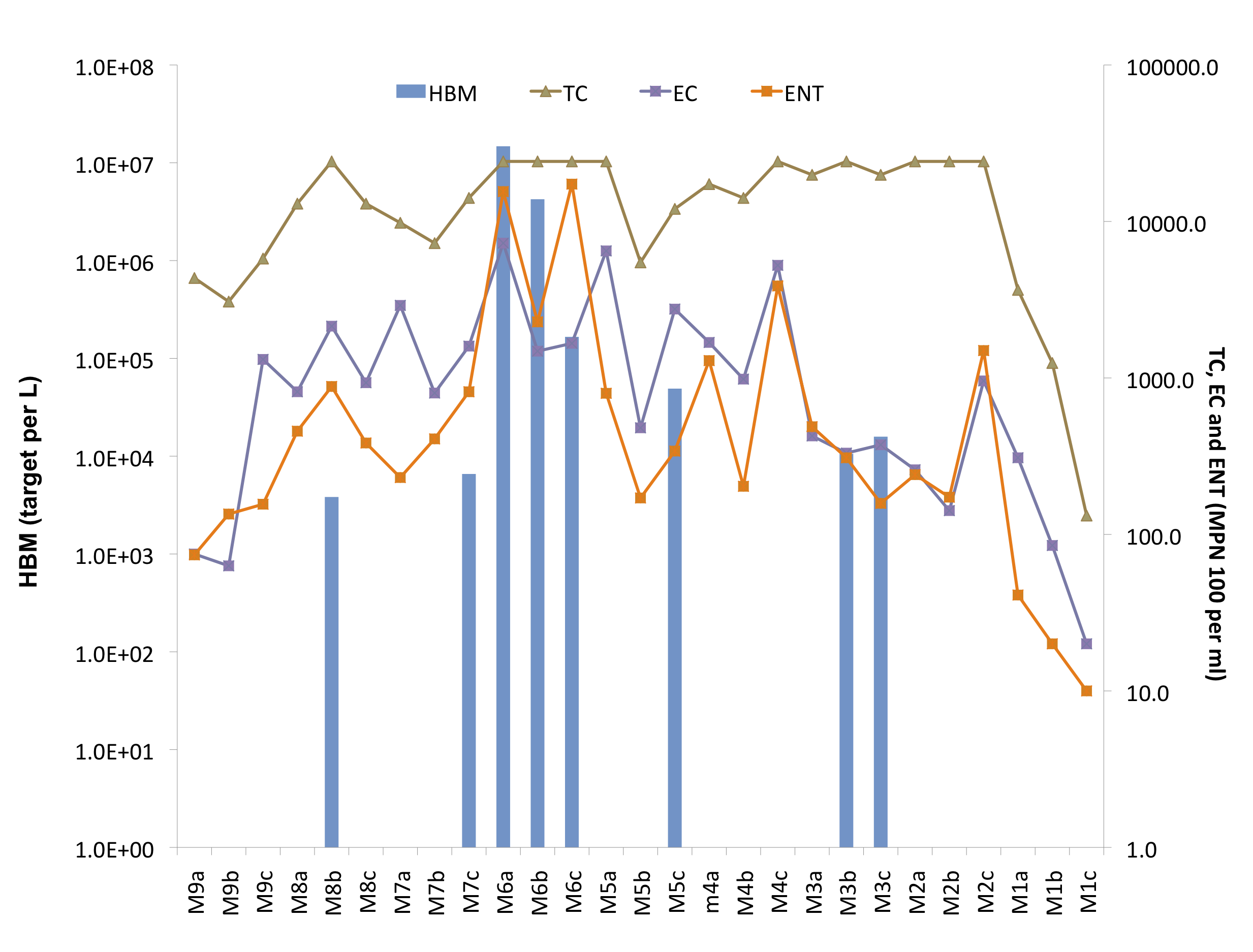

Supplement: Figure S3 — Measurements of Human-specific Bacteroides Marker (HBM), Total Coliform (TC), E. coli (EC), and Enterococcus (ENT) counts. Bars represent HBM values. Lines represent TC, EC and ENT most probable number (MPN). (0.49 MB TIF) [file pone.0011285.s003.tif]

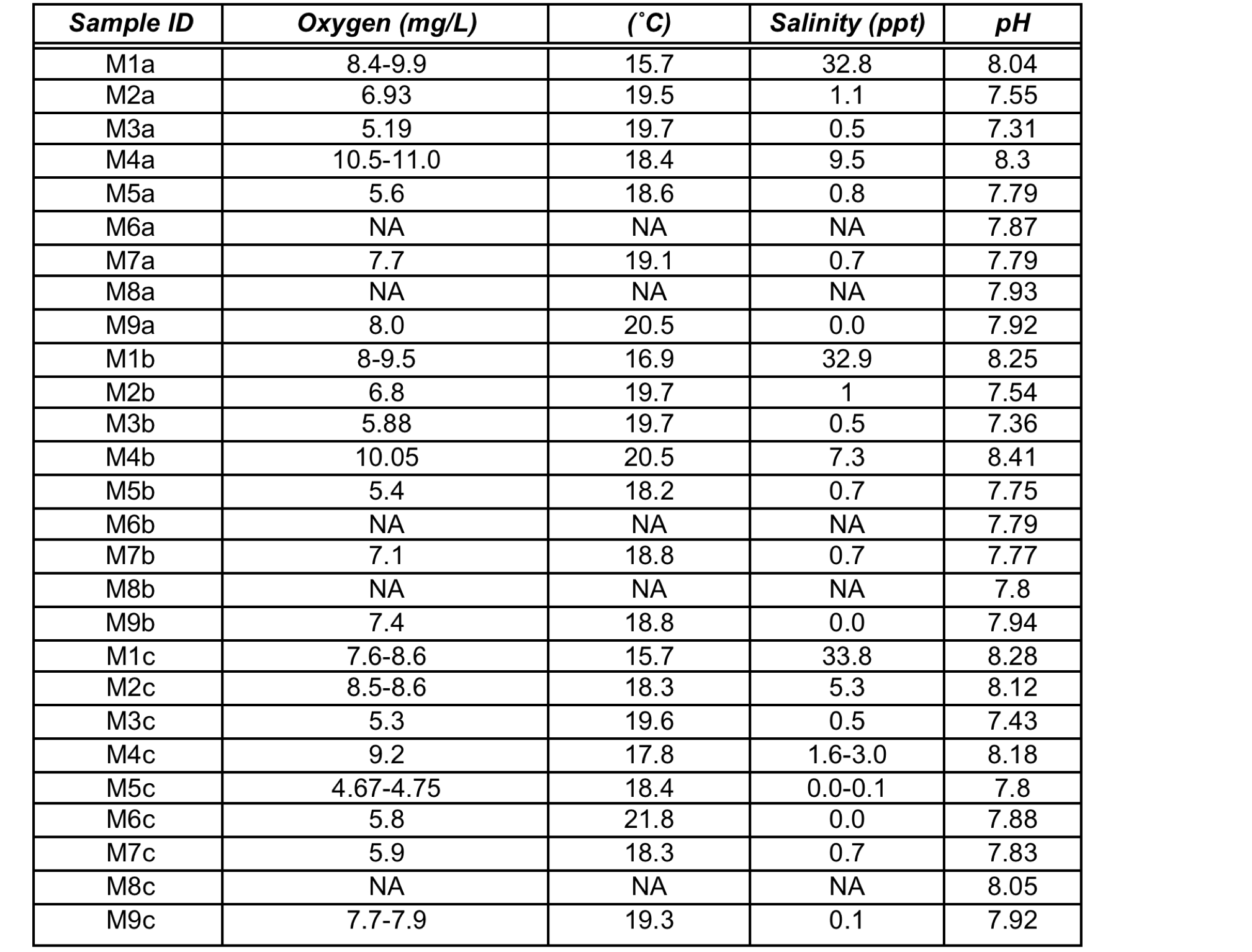

Supplement: Table S1 — Environmental variables measured concurrently with the bacterial community samples. Dissolved oxygen, temperature, salinity and pH were measured at the time of sampling and reported here. (0.42 MB TIF) [file pone.0011285.s004.tif]

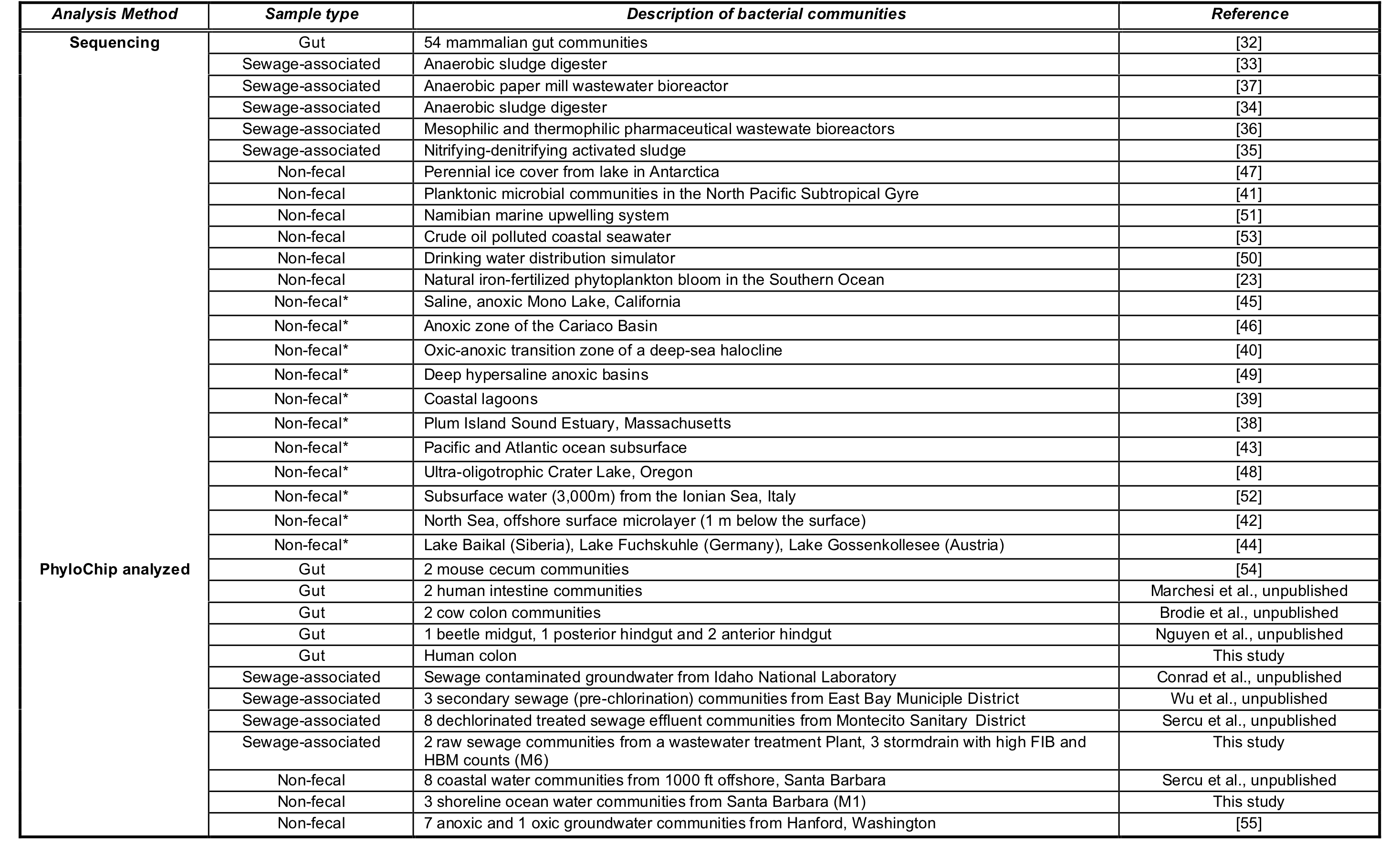

Supplement: Table S2 — Description of bacterial communities analyzed by sequencing and PhyloChip used in Figure 6. Gut, sewage-associated and non-fecal samples analyzed by clone-library sequencing and PhyloChip used for the Bacilli, Bacteroidetes, Clostridia to α-proteobacteria ratio (BBC∶A ratio) are described. All DNA sequences from sequencing samples had a minimum length of 1250 base pairs, except for those with the (*) symbol where the minimum sequence length was 200 base pairs. (0.90 MB TIF) [file pone.0011285.s005.tif]

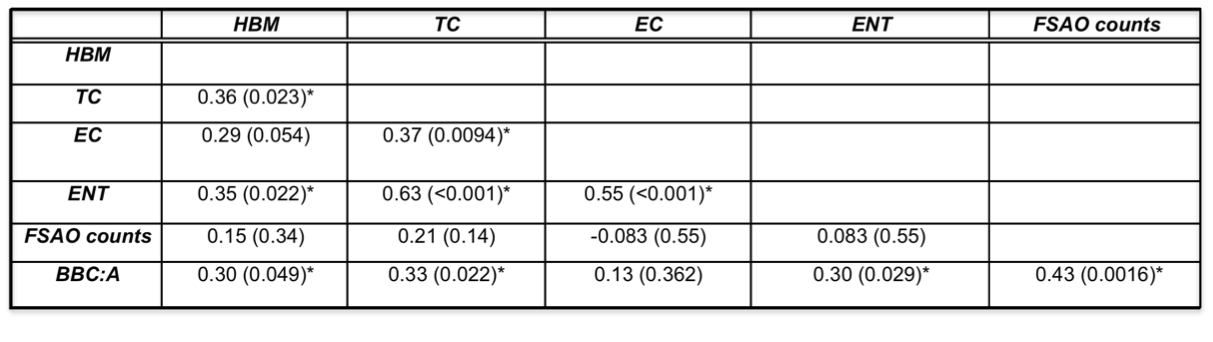

Supplement: Table S3 — Kendall rank correlation tau coefficient and p-values (in parenthesis). Measurements from all 27 water samples were used. The (*) symbol denotes statistical significance (p-value<0.05) differences. Abbreviations: Human Bacteroides Marker (HBM); total coliform (TC); E. coli (EC); enterococcus (ENT); fecal-sample associated OTUs (FSAO); Bacilli, Bacteroidetes, and Clostridia to α-proteobacteria ratio (BBC∶A). (0.09 MB TIF) [file pone.0011285.s006.tif]
